# An update on stem cell and stem cell-derived extracellular vesicle-based therapy in the management of Alzheimer’s disease

**DOI:** 10.1016/j.heliyon.2023.e17808

**Published:** 2023-06-29

**Authors:** Madhan Jeyaraman, Ramya Lakshmi Rajendran, Sathish Muthu, Naveen Jeyaraman, Shilpa Sharma, Saurabh Kumar Jha, Purushothaman Muthukanagaraj, Chae Moon Hong, Lucas Furtado da Fonseca, José Fábio Santos Duarte Lana, Byeong-Cheol Ahn, Prakash Gangadaran

**Affiliations:** a Department of Orthopaedics, ACS Medical College and Hospital, Dr MGR Educational and Research Institute, Chennai, Tamil Nadu, 600056, India; b Department of Biotechnology, School of Engineering and Technology, Sharda University, Greater Noida, Uttar Pradesh, 201310, India; c Indian Stem Cell Study Group (ISCSG) Association, Lucknow, Uttar Pradesh, 226010, India; d Department of Nuclear Medicine, School of Medicine, Kyungpook National University, Kyungpook National University Hospital, Daegu, 41944, Republic of Korea; e Department of Orthopedics, Government Dindigul Medical College and Hospital, Dindigul, Tamil Nadu, 624001, India; f Department of Orthopedics, Shri Sathya Sai Medical College and Research Institute, Sri Balaji Vidyapeeth, Chengalpet, Tamil Nadu, 603108, India; g Department of Paediatric Surgery, All India Institute of Medical Sciences, New Delhi 110029, India; h Department of Internal Medicine & Psychiatry, SUNY-Upstate Binghamton Clinical Campus, Binghamton, NY, 13904, USA; i Department of Orthopedics, The Federal University of São Paulo, São Paulo, 04023-062, SP, Brazil; j Department of Orthopedics, The Bone and Cartilage Institute, Indaiatuba, 13334-170, SP, Brazil; k BK21 FOUR KNU Convergence Educational Program of Biomedical Sciences for Creative Future Talents, Department of Biomedical Sciences, School of Medicine, Kyungpook National University, Daegu, 41944, Republic of Korea

**Keywords:** Alzheimer’s disease, Cellular therapy, Mesenchymal stem cell, Extracellular vesicles, Clinical trial

## Abstract

Globally, neurological diseases pose a major burden to healthcare professionals in terms of the management and prevention of the disorder. Among neurological diseases, Alzheimer’s disease (AD) accounts for 50%–70% of dementia and is the fifth leading cause of mortality worldwide. AD is a progressive, degenerative neurological disease, with the loss of neurons and synapses in the cerebral cortex and subcortical regions. The management of AD remains a debate among physicians as no standard and specific “disease-modifying” modality is available. The concept of ‘Regenerative Medicine’ is aimed at regenerating the degenerated neural tissues to reverse the pathology in AD. Genetically modified engineered stem cells modify the course of AD after transplantation into the brain. Extracellular vesicles (EVs) are an emerging new approach in cell communication that involves the transfer of cellular materials from parental cells to recipient cells, resulting in changes at the molecular and signaling levels in the recipient cells. EVs are a type of vesicle that can be transported between cells. Many have proposed that EVs produced from mesenchymal stem cells (MSCs) may have therapeutic promise in the treatment of AD. The biology of AD, as well as the potential applications of stem cells and their derived EVs-based therapy, were explored in this paper.

## 1 Introduction

Globally, an epidemiological shift from infectious/communicable to non-infectious/non-communicable diseases has been observed which poses a greater burden on healthcare professionals [1,2]. Among the non-communicable diseases, neurological diseases pose a major challenge and threat to both the health care workers and the patients [3,4]. Alzheimer’s disease is responsible for 50%–70% of all cases of dementia and ranks as the fifth leading cause of death globally [3,5]. It is expected that the global incidence of Alzheimer’s disease (AD) will increase to reach a population of 152 million by 2050 [6].

AD is a progressive degenerative neurological disorder with the loss of neurons and synapses in the cerebral cortex and certain subcortical regions leading to cerebral atrophy in the temporal, parietal, and part of the frontal lobe [[7], [8], [9]]. Theories underlying Alzheimer’s disease (AD) include the cholinergic mechanism, protein misfolding, and amyloid cascade hypotheses [10]. The pathognomonic feature of AD is the deposition of amyloid (Aβ) peptides in the extracellular matrix (amyloid plaques), the presence of neurofibrillary tangles (hyperphosphorylated tau protein) in the cortex, amygdala, and hippocampus, neuronal loss, neuroinflammation, and oxidative stress [8,11]. Tauopathy is strongly correlated with functionally deficit AD brains [12]. The abnormal hyperphosphorylation leads to loss of tau function of promoting assembly and stabilizing microtubules and procurement of toxic function thus the pathological tau sequesters normal tau, MAP-1A/-1B and -2, and reasons disruption and inhibition of microtubules [13]. Tau pathology in AD follow neuroanatomical pathways which reflect transmission of abnormal tau protein from cell to cell in a “prion-like” fashion [14]. The cognitive impairment severity correlates best with the burden of neocortical neurofibrillary tangles which are produced by abnormal tau proteins.

The National Institute of Neurological and Communicative Disorders and Stroke (NINCDS) has established diagnostic criteria for Alzheimer’s disease (AD), which include: a) the existence of cognitive deficits (such as memory loss, language difficulties, perceptual problems, and disorientation), and b) the verification of a probable dementia diagnosis through neuropsychological assessment [15,16]. The literature does not currently propose a standardized treatment approach for Alzheimer’s disease (AD). However, a primary treatment strategy for reversing AD involves decreasing Aβ levels [[17], [18], [19]]. Researchers worldwide have conducted significant investigations aimed at comprehending the pathogenesis and natural progression of Alzheimer’s disease (AD), as well as improving its diagnosis and exploring regenerative principles for its management [[20], [21], [22], [23], [24]]. While there is ongoing research into developing “disease-modifying” treatments to halt or reverse the underlying pathophysiological mechanisms of Alzheimer’s disease (AD), no specific treatment has been identified yet. Given the complexity of AD’s pathogenesis, a multimodal approach to treatment is recommended, involving pharmacological therapy, behavioral interventions, and the stimulation of neurogenesis and synaptogenesis through endogenous or exogenous means. This paper reviews recent research advancements, from experimental to clinical studies, in the use of stem cells and stem cell-derived extracellular vesicles (EVs) for the treatment of AD.

## 2 Stem cells and Alzheimer’s disease (AD)

**Fig. 1:**
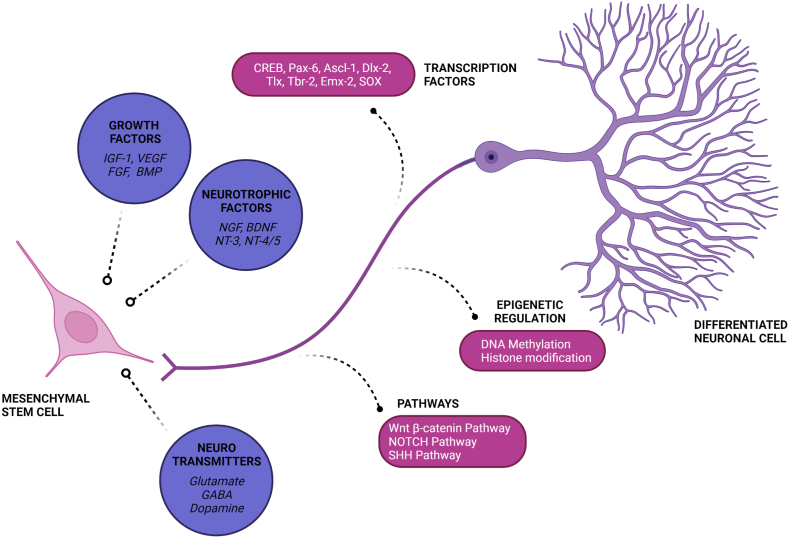
Neurogenic signaling of adult stem cells mediated through growth factors, neurotrophic factors, and neurotransmitters as shown in circles. The pathways involved in their signaling and their mediation of action through epigenetic regulation and transcriptional factors involved are given in boxes. Created with BioRender.com

At this time, there is no documented permanent cure for AD. The use of medications to treat AD temporarily improves cognitive symptoms. Researchers are concentrating their efforts on three specific areas of AD research: a) anti-oxidation, b) removal of Aβ deposits from the brain and c) regulation of the phosphorylation of tau protein [10,[25], [26], [27], [28], [29]]. The emergence of regenerative and translational medicine has prompted a growing interest in the potential of computed tomography scans and cell-based replacement therapies for the development of disease-modifying treatments for Alzheimer’s disease (AD). Stem cells, which are undifferentiated, blank cells, have the ability to differentiate and proliferate into neural cells when activated by signaling pathways, making them a promising tool for replenishing lost neuronal cells and restoring neuroplasticity and neuromodulation in AD [30,31]. Neurogenic signaling for adult stem cells is mediated through various pathways are shown in Fig. 1. In the last decade, there has been mounting evidence to support cell-based neuron replacement therapy for AD in both pre-clinical and clinical trials. Table 1 lists the results of stem cell therapy in an animal model of AD.

**Table 1:** Stem cell-based therapies in AD animal models.

Types of Stem Cells Used	Animal Model Used	Route of administration	Results/Outcome
Neural stem cells [32]	Triple transgenic mice (3xTg-AD) that express PS1, tau, and APP	Intrahippocampal injection	Ameliorated loss in spatial memory and learning by BDNF Increase in synaptic density
Neural precursor cells [33]	Focal cerebral ischemia in the rat model	Intranasal	Enhanced neurogenesis due to the cross-talk between NPCs and the resident endogenous cells
Umbilical cord-derived MSCs (UC-MSCs) [34]	Double transgenic mice of PS1 and APP	Intracerebral implantation	Enhanced spatial learning and memory with modulation of neuroinflammation
Transdifferentiated human Wharton’s jelly-derived MSCs into neuron-like cells [35]	AβPP/PS1 transgenic mice model	Intracerebral implantation	Enhanced cognitive functions with decreased Aβ load
Clinical grade MSCs [36]	Amyloid β treated mice	Intracerebral implantation	Potentiated neurogenesis in the hippocampus with NPCs differentiation
Bone marrow-derived MSCs (BM-MSCs) [37]	APP/PS1 transgenic mice	Intravenous administration	Reduction of Aβ plaques
Human olfactory bulb neural stem cells [38]	Ibotenic acid-induced AD rat model	Intrahippocampal injection	Nerve growth factors expression by engineered stem cells with potentiation of cognition
Placenta-derived MSCs [39]	Amyloid β1-42 peptide infused in a mouse model	Intracerebroventrical (ICV) infusion	Differentiation of neuronal cells with enhanced cognitive function
VEGF overexpressing BM-MSCs [40]	A dual transgenic (APPswe/PS1dE9 mutations) mouse model	Intracerebroventrical (ICV) infusion	Improved behavioral responses with reduced Aβ plaque load; Enhanced vasculogenesis
Choline acetyltransferase expressing human NSC [41]	AF64A-cholinotoxin-induced learning deficit rat model	Intracerebroventrical (ICV) infusion	Differentiated neurons migrate toward the injured site
Epidermal neural crest stem cell [42]	Amyloid-β1-40 injected AD rat model	Intrahippocampal injection	Augmented granule cells in the hippocampus; Transplanted cells expressed neuronal markers (β-tubulin and MAP2)

Neural stem cells (NSCs), also referred to as neurogenic precursor cells (NPCs), are found in limited areas of the adult brain that exhibit ongoing neurogenesis and neuromodulation. The activation and enhancement of NSCs during embryonic and adult neurogenesis, synaptogenesis, proliferation, migration, and differentiation of NPCs are facilitated by neurogenic signaling molecules [[43], [44], [45]]. Neural stem cells (NSCs) that have been genetically modified can be utilized to deliver neurotrophic factors that enhance gene expression, potentially modifying the natural progression of Alzheimer’s disease (AD) [46,47].

### 2.1 Embryonic stem cells (ESCs)

Embryonic stem cells (ESCs) are totipotent cells that can self-renew and differentiate into a variety of cell types. ESCs are formed from the inner cell mass of the blastocyst, which is responsible for the development of the entire organism. Because of their pluripotent nature, ESCs have the potential to cause cancer, unregulated cellular proliferation, and immunogenic rejection [48]. Tang et al. proposed the hypothesis that transplantation of embryonic stem cells (ESCs) from the hippocampus could restore cognitive function in rats with Aβ peptide-induced damage without any associated risks [49]. In a rodent model of AD, the transplantation of mouse ESC-derived neural progenitor cells (NPCs) and/or the commitment of these cells to a cholinergic cell phenotype can improve cognitive and behavioral recovery, as well as the cholinergic neurons regeneration [50]. An AD model was given a boost in learning and memory abilities after NPCs generated from mouse ESCs were transplanted into the basal nucleus. When basal forebrain cholinergic neurons generated from mouse and human ESCs were implanted into the transgenic AD mouse model, an improvement in neurocognitive recovery was seen. A total of human ESCs were shown to be capable of developing into astroglial cells, spinal motor neurons, and dopaminergic neurons [51]. NPCs generated from ESCs develop into astrocytic and neuron-like cells and improve learning and memory function in AD animal models [49,52]. ESCs used in FDA-approved clinical trials, on the other hand, raise ethical questions due to the invasiveness of procuring ESCs [53,54].

### 2.2 Mesenchymal stem cells (MSCs)

MSCs show a significant role in a) immunoregulation, b) neurotrophism, c) neuroplasticity, and d) the decrease of Aβ plaque burden to restore cognitive function in AD [55,56]. Several preclinical investigations have revealed that pathogenic alterations in AD animal models can be reversed. These investigations confirmed that MSCs had neuroprotective, neuroplastic, neuromodulatory, and neurogenic effects through the activation of neurogenic signaling pathways in the brain [57]. The transplanted MSCs boost the expression of anti-apoptotic factors, help to maintain working memory, and decelerate the progression of the disease [56,58]. The regulation of microglial activation and the reduction of Aβ plaque levels in the brain are responsible for the restoration of cognition mediated by MSCs [59]. The anti-inflammatory and immunomodulatory properties of MSCs promote neuroprotection while suppressing inflammation in the body. By targeting Aβ deposition with miRNAs and siRNAs, MSCs release extracellular vehicles into the environment [60].

Increased astrocytosis and synaptogenesis in AD model mice following intracerebroventricular injection of BM-MSCs have been shown to alleviate cognitive impairment in the mice [61]. The hippocampus of animals treated with BM-MSCs has higher levels of miR-146, an exosome that promotes synaptogenesis and neurogenesis while also restoring neuroplasticity and cognitive impairment [62]. In a rat model of Alzheimer’s disease (AD), intravenously injected BM-MSCs were observed in the brain parenchyma within 1 h of injection, releasing growth factors and cytokines that facilitate the restoration of neurobehavioral functions and stimulate endogenous regeneration within the brain [63]. Intravenous injection of MSCs in young and old 3xTg-AD mouse models of AD display reduced tau phosphorylation and neuroinflammation. The timing and the frequency of MSC injection demonstrate a neuroprotective effect on the 3xTg-AD mouse model of AD [64]. Transplantation of bone marrow-derived MSCs improves cognitive deficits by reducing Aβ protein and tau aggregates, decreasing intercellular tau hyperphosphorylated aggregates and tau tangles, mitigating apoptosis due to aberrant protein clearance, and inhibiting neuroinflammation in AD [65].

In a mouse AD model, adipose tissue-derived MSCs (AD-MSCs) improved neural function, increased neurogenesis, and differentiated into neuron-like and astrocyte-like cells. After 12 days of injection, AD-MSCs were discovered in the brains of animals who had received intravenous delivery of the cells. Increased neurogenesis and decreased Aβ plaque depositions are observed following the transplantation of AD-MSCs into the mouse brain. These improvements are associated with improved cognition and memory abilities [66]. After receiving A1–42 treatment, mice were given an intracerebroventricular transplant of placenta-derived MSCs (PD-MSCs), which revealed an inhibitory effect on neuronal death and memory impairment [39]. In the brain, PD-MSCs potentiate the production of anti-inflammatory cytokines. They encourage proliferation in the hippocampus and neuronal development of the AD mice model, which is a positive outcome. The administration of MSCs to elderly AD rats results in the restoration of motor and cognitive activity [58]. Preclinical experiments using MSCs in AD have yielded positive results. Nine patients participated in a phase I investigation of MSC injection directly in the human brain, which confirmed the possibility and safety of injection of MSCs directly in the human brain in one human trial on MSCs in AD [[67], [68], [69]].

### 2.3 Neural stem cells (NSCs)

Due to the multipotent nature of neural stem cells (NSCs), when they are transplanted, they differentiate into neurons, astrocytes, and oligodendrocytes, making them the ideal cell-based therapy for neurodegenerative illnesses. NSCs are derived from primary tissues such as the fetus, postmortem neonatal or adult brain tissues, ESCs, and induced pluripotent stem cells (iPSCs) [31,53,70]. Direct separation of NSCs from primary tissue, on the other hand, is exceedingly dangerous, because non-patient-specific NSCs have the potential to provoke immunological rejection. NSCs in the mammalian brain are involved in neuronal homeostasis, repair, and regeneration, as well as pleiotropism (the ability to respond to different stimuli) [[71], [72], [73]].

Transplantation of NSCs in combination with neurotrophic agents resulted in neuronal cells and glial cells differentiation [74]. Neurotrophic factors are secreted by the brain and cause prolonged neurogenesis in the zones of sub-granular and sub-ventricular, this improves memory due to its paracrine actions [75]. It has been discovered that engineered NSCs may increase the production of the Aβ degrading enzymes such as neprilysin (NEP), endothelin-converting enzyme, and insulin-degrading enzyme, resulting in less Aβ aggregation and an increase in the density of synapses [76].

In the primates' AD model, NSCs enhance neurogenesis and synaptogenesis along with the reduction of Aβ plaques and phosphorylation of tau protein and potentiate motor, cognition, spatial learning, and memory [76,77]. Transplanting NSCs in of AD animal models can help to restore damaged neuronal circuits by secreting neurotrophic factors, leading to a reduction in the amount of Aβ plaques within the brain. Additionally, NSC transplantation increases the number of cholinergic neurons in the brain, thereby enhancing learning and memory functions [76]. It remains uncertain whether the observed improvements in behavior, memory, and learning capabilities in disease models of AD following NSC transplantation are a result of NSC differentiation or the neurotrophic factors they secrete [76]. Transplanted neural stem cell grafts raise brain-derived neurotrophic factor levels and improve behavioral recovery in a mutant hAPP-overexpressing mouse model of AD without affecting Aβ or tau pathology [78].

In an APP/PS1 transgenic AD mouse model, intranasal transplantation of human NSCs results in the differentiation of cholinergic neurons, which reduces amyloid accumulation by upregulating amyloid degrading enzymes, and neprilysin expression reduce neuroinflammation, pericytic and synaptic loss, and restores cognitive function. The use of human NSCs for intranasal transplantation in the treatment of AD has the greatest potential as a non-invasive treatment option that reduces Aβ levels in animal models of AD by inhibiting the activity of cathepsin B, plasmin, and insulin-degrading enzymes [79]. Human-induced neural progenitor cells (iNPCs) were transplanted into the hippocampus of immunodeficient mouse model of AD, where they proved long-term survival, formation of graft-host synaptic connections, establishment of neuronal circuits and regeneration of the neural network within the host hippocampus. Mice with AD showed signs of neuroplasticity, neuromodulation, and synaptogenesis, as well as improvements in their cognitive and behavioral abilities [80].

The invasion of NSCs into the fimbria and the fornix junction in the APP/PS1 mouse model of AD results in a considerable improvement in cognition and memory capacities. The activation of microglia and the phagocytosis of amyloid plaques are induced by NSCs [81]. Transplantation of NSCs to the hippocampus does not achieve the intended benefits in the mouse AD model when the microenvironment is not first primed with neurotrophic factors [82]. The administration of NGF to elderly rats revealed an improvement in cognition [82]. In the phase 1 clinical experiment, fibroblasts (genetically modified) programmed with the gene of NGF were proven to be safe when transplanted into the forebrain (NGF-gene therapy for AD) [83]. In 2015, Tuszynski et al. reported trophic response to NGF gene therapy in AD in the form of axonal sprouting and neurons exhibit tau free expression of NGF indicating the therapeutic genes leads to activation of cell signaling in AD [84]. NGF gene biodelivery maintain long term cognitive efficiency in cases with AD [85,86]. These findings point the way forward and open up a new vista in the treatment of AD in humans.

### 2.4 Induced pluripotent stem cells (iPSCs)

Yahata et al. and Yagi et al. were the initial researchers to use iPSCs to treat AD in 2011 [87,88]. Yahata et colleagues demonstrated that the transplantation of iPSCs derived from forebrain neurons might reduce the development of Aβ plaques in neuronal cultures [88]. Yagi et al. revealed that the PS1 (A246E) and PS2 (N141I) mutations result in increased A42 secretion while attenuating the pathogenic mechanism of familial AD [87]. Neurons and neurospheres can be distinguished from iPSCs. It has been shown that direct injection of iPSCs into the mouse brain leads to the production of neuronal progenitors. The iPSCs treatment of mouse models with AD led to increased neprilysin, an Aβ-degrading protease expression in the cells [89]. Synaptic activity and connectivity between excitatory and inhibitory neurons are enhanced in neurons produced from iPSCs [90,91]. Purified juvenile neuronal cells were converted into mature neurons after being cultured for an extended period. The fully mature neurons were found to have both excitatory glutamatergic and inhibitory GABAergic connections, and the ability to enhance action potential potentiation. Studies have demonstrated that cortical neuronal cells produced from human-iPSCs can decrease the formation of Aβ plaques and tau protein complexes in the forebrain [92].

The treatment of AD models using iPSCs produced from neuronal cells from an AD donor resulted in the downregulation of the tau protein. The transcriptome study of the iPSC-derived neuronal cells revealed that the expression of genes related to AD had changed significantly [93]. The 5XFAD transgenic AD mice model exhibited improved cognitive abilities, along with the differentiation of protein-iPSCs into glial cells. Furthermore, the iPSC-derived cells were shown to reduce the accumulation of Aβ plaques and diminish their size. When protein-iPSCs are injected into the brain, the researchers discovered an increase in oligodendrocyte-related genes, which they confirmed using proteomic analysis [94]. A 3D spheroid derived from human iPSCs for the AD model was developed into neuronal cell lines, which resulted in a reduction of both A40 and A42 levels and, as a result, increased the ability of the cell lines to regenerate back into neuronal cells [95]. In order to mimic the architecture of the human brain, including the cerebral progenitors found in the zones of sub-ventricular and sub-granular in the hippocampus, 3D cerebral organoids are utilized within a three-dimensional environment [96]. Compared to MSCs, human-iPSCs generate exosomes at a rate that is 16 times higher. These exosomes possess an enhanced ability to reduce the accumulation of Aβ plaques in the brain and have been demonstrated to increase the A42/A40 ratio in animal models of Alzheimer’s disease [96].

## 3 Stem cell-derived extracellular vesicles (EVs)-Based therapy for AD

**Fig. 2:**
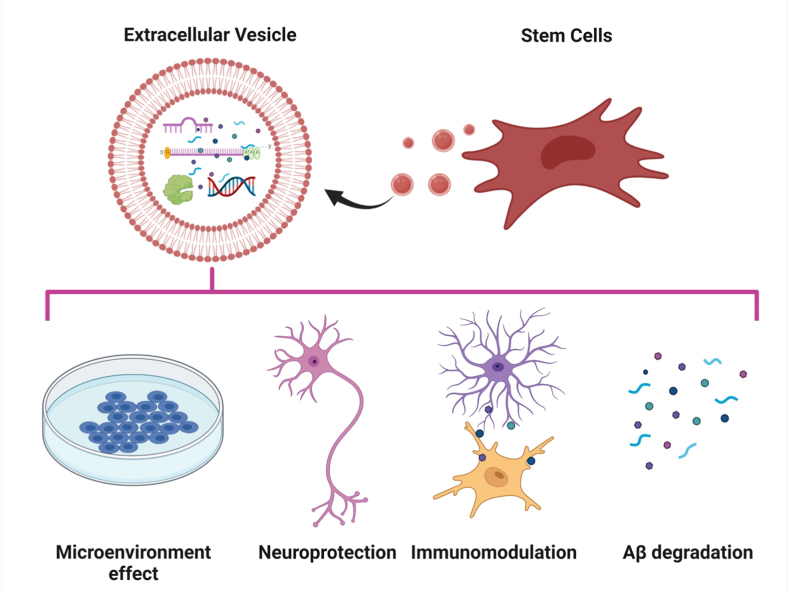
Actions of MSC-EVs in AD. Created with BioRender.com

As a prospective and viable alternative to cell-based therapies in the field of regenerative medicine, ESC-derived EVs are being studied. Extracellular vesicles (EVs) or exosomes are naturally released by all eukaryotic cells through the shedding of plasma membrane, serving to promote the paracrine effect and preserve tissue homeostasis. The biogenesis of these vesicles commences with the internal budding of the endosomal membrane [[97], [98], [99]]. In this process, specific proteins such as EV cargo, as well as some cytosolic contents, are incorporated, forming intraluminal vesicles (ILV) within multivesicular bodies (MVB). The MVBs can follow one of two fates - either the direct secretion of the ILVs into the extracellular matrix (ECM) by fusing with the plasma membrane, or fusing with lysosomes for the degradation of the ILVs and their contents. The significant increase in exosome secretion from cancer cells suggests a shift towards the release of exosomal cargo, rather than lysosomal degradation [100]. This type of shift may also occur in non-transformed cells such as antigen-presenting cells, they release greater quantities of exosomes upon stimulation [101]. In recent research, it has been proposed that the therapeutic actions of stem cells in AD are mostly mediated by secretory factors, such as EVs as shown in Fig. 2 [102]. Many research have revealed the therapeutic potential of ESC-derived EVs in the treatment of AD (Table 2). According to the information available, stem cell-derived EVs are being evaluated as a potential treatment strategy for AD. An in vitro model of AD has been established using exosomes derived from adipose-derived stem cells (ADSC-Exo). The therapeutic potential of ADSC-Exo has been investigated for its ability to prevent disease phenotypes induced by the Aβ cascade in NSCs derived from the brains of TG2576 AD mice. Compared to control cells, ADSC-Exo treatment revealed lower Aβ levels and a lower Aβ 42/40 ratio, normalized the increased Bax, p53, and caspase-3 levels, and reduced Bcl-2 levels, resulting in less apoptosis in AD model cells and increased neurite outgrowth in NSCs. These findings imply that ADSC-Exo may have the potential as a therapeutic tool for the treatment of Aβ-induced neuronal death and AD [103]. A study investigated the influence of MSC-derived EVs on the neuroprotective capacity of BM-MSCs. Through the prevention of the loss of pre-and postsynaptic markers, MSC-EVs protect neurons against AO-induced oxidative stress and synapse degradation in the brain. According to the findings of this investigation, MSC-EVs could be a promising option or addition to cell-based therapies in the treatment of AD [104].

**Table 2:** Effects of Stem cells derived EVs in AD.

EVs source	Model	Culture or Route of administration	Outcome
ADSCs [103]	*In vitro*	Co-culture with NSCs	Reduced Aβ levels, reduced cell apoptosis and increased neurite growth.
BM-MSCs [104]	*In vitro*	Co-culture with neurons	Prevent the loss of synapses in neurons exposed to Aβs.
ADSCs [105]	AD mice	Intranasal	Reduced neuronal toxicity, increased newborn neurons, and rescued memory.
Wharton’s jelly MSCs [106]	AD mice	Intravenous	Reduced expression of Aβ and restored memory, glucose metabolism, and cognitive function.
BM-MSCs [107]	AD mice	Intravenous	Reduced deposition of Aβ and improved recovery of cognitive function
BM-MSCs [108]	AD mice	Intracranial	Reduced burden of Aβ Plaque in early stages of AD
AD-MSCs [109]	AD mice	Intravenous	Increased nerve function, motor ability, and survival level of nerve cells and decreased the inflammatory factors
BM-MSCs [110]	AD mice	Intravenous	Rescue memory deficits by regulating inflammatory responses
BM-MSCs [111,112]	AD mice	Intranasal	Enhances immunomodulatory and neuroprotective effects

ADSC-EVs have been employed to investigate the therapeutic potential of the cells in preventing the disease phenotypes in an in vivo AD model. The administration of ADSC-EVs by intranasal injection into AD mice revealed that they entered the brain swiftly and efficiently accumulated in neurons. Treatment of ADSC-EVs with neuroprotective and neurogenesis proteins has been demonstrated to result in neuroprotection and neurogenesis, with the genes involved in neuroprotection being enriched in the treated cells. ADSC-EVs therapy also reduced neuronal toxicity and neurologic impairment in the whole-brain areas of AD mice. The researchers discovered that they could boost newborn neurons and reverse memory deficiencies in APP/PS1 transgenic mice, which was significant. These data imply that ADSC-EVs can help to reduce neuronal damage, induce neurogenesis, and restore memory loss in the AD mouse model [105]. Exosomes derived from Wharton’s jelly-derived MSCs (MSC-Exo) were tested for their potential therapeutic effects in AD. A human neural cell culture model with familial Alzheimer’s disease (FAD) mutations responded favorably to MSC-Exo therapy, which decreased Aβ expression while increasing the expression of neuronal memory/synaptic plasticity-related genes. A significant improvement in brain cognitive and function glucose metabolism were observed in the AD model following MSC-Exo treatment (intravenous infusion of MSC-Exo). 18F-FDG-PET imaging and cognitive assessment were used to evaluate the results of the study. It has also been demonstrated that MSC-Exo can modulate the phase of neurons and astrocytes in the brains of AD mice. As a result of these observations, MSC-Exo may have therapeutic promise in the treatment of AD [113].

Using BM-MSC-derived exosomes, researchers explored the role and mechanism of exosome signaling in AD. AD mice were given an intravenous injection of MSC-Exo therapy, which improved spatial learning and memory skills while also increasing the expression of sphingosine kinase (SphK) and sphingosine-1-phosphate (S1P) in the brains of the animals. In addition, MSC-Exo therapy reduced the levels of amyloid in the brains of AD mice and increased the expression of NeuN in the cortex and hippocampus. In addition, MSC-Exo therapy decreased the levels of A140, A142, and BACE1, as well as the expression of neprilysin, and increased the expression of neprilysin. These findings imply that lowering Aβ deposition and increasing cognitive function recovery in mice can be accomplished through a combination of strategies [107]. EVs generated from BM-MSCs were employed to examine the therapeutic effect of EVs in the AD mouse model. It was discovered that injecting MSC-EVs into the neocortex of AD mice reduced the amount of Aβ plaque in the mice’s brains. Additionally, the amounts of dystrophic neurites were lowered (cortex and hippocampus). As a result of these findings, BM-MSC-EVs may play a role in the early phases of AD [108].

The discovery that miRNA-22 can suppress pyroptosis by targeting GSDMD leads to improvements in the memory and motor ability of mice with AD was made previously. This was the situation in which the same group transfected AD-MSCs with a miRNA-22 mimic to obtain exosomes containing miRNA-22 (Exo-miRNA-22). The administration of Exo-miRNA-22 to AD mice resulted in improved nerve function, motor ability, and nerve cell survival levels, as well as a reduction in inflammatory markers. In vitro cultivation with A25-35-induced PC12 cells treated with Exo-miRNA-22 revealed that the cells were less susceptible to apoptosis and secreted fewer inflammatory factors [109]. Using a BM-MSC-derived exosome, another study targeted the brain of AD mice using a tagged rabies virus glycoprotein (RVG) peptide that was unique to the central nervous system (MSC-RVG-Exo). Intravenous administration of MSC-Exo or MSC-RVG-Exo into AD mice leads to reduced plaque deposition. Additionally, the activation of astrocytes was reduced, and cognitive performance was improved in comparison to MSC-Exo. Furthermore, MSC-RVG-Exo treatment was found to be more effective than MSC-Exo treatment in reducing the expression of pro-inflammatory mediators (TNF-a, IL-a, and IL-6) in the brain [110]. The therapeutic effects of EVs produced from BM-MSCs in the AD mouse model were investigated. MSC-EVs were delivered intranasally into AD mice, resulting in immunomodulatory and neuroprotective benefits in the mice. Additionally, it reduced the activation of microglia cells and increased the density of dendritic spines [111]. Using tiny EV (MSC-sEV) generated from BM-MSCs, researchers explored the role and mechanism of action of MSC-sEV in AD. AD mice received an intranasal dose of MSC-sEV therapy, which demonstrated dramatically improved behavior in cognitive tests and decreased Aβ plaque load in the hippocampus. These findings imply that MSC-sEV can reduce the progression of AD [112]. Based on the findings of the aforementioned research in AD, MSC-derived EVs have the potential to be used as a cell-free therapeutic tool and as a drug delivery vehicle.

## 4 Clinical trials on AD

We looked into the active trials on cellular therapy (CT) for AD and discovered 16/18 trials looking into the potential of various sources of MSCs, one of which looked into the potential of MSC-derived exosomes, and one looking into the potential of iPSCs for AD [114]. It is undeniable that MSCs appear to be the most favored and potentially available source of CT for AD, with few ethical limits on its use for the disease. The United States, Korea, and China are leading the way in CT research and clinical trials for AD. Even though the results of prior tests with MSC-EVs have been promising, only one clinical study is now underway or has been completed that has investigated the possible function of MSC-exos in clinical trials. (Table 3).

**Table 3:** Clinical trials of Stem cells and stem cell-derived EVs in patients with AD.

Sl.No	NCT Number	Title	Status	Interventions	Locations
1	NCT02833792	Allogeneic Human Mesenchymal Stem Cells for Alzheimer’s Disease	Recruiting	BM-MSCs	USA
2	NCT04855955	Autologous Human Adipose-Derived Mesenchymal Stem Cells in Alzheimer’s Disease	Available	AD-MSCs	USA
3	NCT02600130	Allogeneic Human Mesenchymal Stem Cell Infusion Versus Placebo in Patients With Alzheimer’s Disease	Active, not recruiting	BM-MSCs	USA
4	NCT04040348	Alzheimer’s Disease Stem Cells Multiple Infusions	Recruiting	BM-MSCs	USA
5	NCT02672306	Safety and Exploratory Efficacy Study of UCMSCs in Patients With Alzheimer’s Disease	Unknown status	UC-MSCs	China
6	NCT03724136	Alzheimer’s Autism and Cognitive Impairment Stem Cell Treatment Study	Recruiting	BM-MSCs	UAE
7	NCT01547689	Safety and Efficiency of Umbilical cord-derived Mesenchymal Stem Cells(UC-MSC) in Patients With Alzheimer’s Disease	Unknown status	UC-MSCs	China
8	NCT01297218	The Safety and The Efficacy Evaluation of NEUROSTEM®-AD in Patients With Alzheimer’s Disease	Completed	UC-MSCs	Korea
9	NCT02054208	Safety and Exploratory Efficacy Study of NEUROSTEM® Versus Placebo in Patients With Alzheimer’s Disease	Completed	UC-MSCs	Korea
10	NCT04388982	the Safety and the Efficacy Evaluation of Allogenic Adipose MSC-Exos in Patients With Alzheimer’s Disease	Recruiting	MSC Exos	China
11	NCT04482413	Study to Evaluate the Safety and Efficacy of AstroStem in the Treatment of Alzheimer’s Disease	Not yet recruiting	AD-MSCs	USA
12	NCT03117738	A Study to Evaluate the Safety and Efficacy of AstroStem in the Treatment of Alzheimer’s Disease	Completed	AD-MSCs	USA
13	NCT04954534	Exploratory Efficacy Study of NEUROSTEM® in Subjects Who Control Group of NEUROSTEM®	Not yet recruiting	UC-MSCs	Korea
14	NCT03172117	Follow-up Study of Safety and Efficacy in Subjects Who Completed NEUROSTEM® Phase-I/IIa Clinical Trial.	Recruiting	UC-MSCs	Korea
15	NCT01696591	The Long-Term Safety and Efficacy Follow-Up Study of Subjects Who Completed the Phase I Clinical Trial of NEUROSTEM®-AD	Unknown status	AD-MSCs	KOREA
16	NCT03297177	Autologous Stem/Stromal Cells in Neurological Disorders and Disease	Not yet recruiting	ADMSCs	USA
17	NCT04684602	Mesenchymal Stem Cells for the Treatment of Various Chronic and Acute Conditions	Recruiting	ADMSCs	USA
18	NCT00874783	Development of iPS From Donated Somatic Cells of Patients With Neurological Diseases	Recruiting	iPSCs	Israel
19	NCT04388982	The Safety and the Efficacy Evaluation of Allogenic Adipose MSC-Exos in Patients With Alzheimer’s Disease	Recruiting	ADSC-Exos	China

## 5 Challenges & future directives of stem cell and stem cell-derived extracellular vesicle-based therapy in AD

The recent evidence point towards impaired endogenous neurogenesis and a reduction in the neuronal stem cell pool in the hippocampus of patients with AD. Hence MSC transplantation makes replenish such depleted pools to aid in the enhanced neurogenesis as demonstrated in the mice model [115]. There is no other therapeutic intervention that has the pleiotropic effects of MSCs in AD. But the ways to maximize this effectiveness remain a query. Stem cell-based biological scaffolds and microvesicles would stand as an amenable strategy towards a targeted therapeutic strategy for prolonged survival of supplemented MSCs in the central nervous system [[116], [117], [118]]. The recent advances presented in the form of ongoing clinical trials and animal models are evaluating the real-time effectiveness of such treatment methods.

A great deal of effort has been undertaken by clinicians and regenerative experts to bring CT or EVs into clinical practice in the treatment of neurological illnesses. The results of preclinical research conducted on illness models have provided encouraging information regarding the efficacy and safety of these drugs. Clinical trials were also undertaken to determine the efficacy of CT or EVs in the treatment of AD. Despite the good and inspirational advancements in their usability that have occurred in clinical trials, there are still several challenges to overcome before they may be used in routine clinical practice. They must not be disregarded before the implementation of CT or EVs for the treatment of these neurological illnesses in the clinic.

Identifying the proper sort of cell that will provide the most assistance in various neurological illnesses is important. Furthermore, an improved understanding of their treatment mechanism should be taken into account. For example, for a CT to be effective in Parkinson’s disease, specific variations of dopaminergic substantia nigra neurons must be present. Hence CT intended for use in the settings described above must be differentiated into several different cell lines. The primary mechanism of CT is differentiation theory and paracrine theory along with immune modulation and enhancement of endogenous neurogenesis, contributing to the therapeutic benefits of the treatment [31,119]. Understanding these effector mechanisms before providing these medications to patients with neurological diseases is therefore critical.

According to the information available thus far, the evidence derived from animal models of neurological illnesses may not entirely reflect all elements of the condition, making their implementation in clinical settings potentially harmful. For starters, the rodent models that are now available do not accurately represent the physiological state of the disease in people. The age of the animals utilized in the models of AD ranges from very young to very old. Second, animal models continue to be insufficient for demonstrating improvements in clinically relevant functional deficits in people with neurological disorders. Third, it is possible that the animal models will not demonstrate all of the essential negative effects of CT.

After that, the evaluation of their tumorigenicity remains a significant difficulty in CT. Patients with neurological illnesses such as Parkinson’s disease (PD) should expect to have a normal lifespan. In such a situation, it would be unbearable to even consider the possibility of tumor growth as a result of CT scanning. To better understand the tumorigenicity of stem cells and their derivatives, it is essential to conduct a large number of studies. It will take the combined efforts of researchers and clinical specialists to avoid the probability of such tragic events and the consequences they will have on the individuals who will be affected by them. Furthermore, such medicines must be sufficiently competitive with traditional therapies in terms of efficacy and safety when compared to the latter.

Furthermore, such stem cell therapies are coupled with questions about ethics as well as the possibility of graft rejection. Because of the poor rate of generation of iPSCs from the patient’s somatic cells, the cost of individualized therapy using stem cell therapies is increased [70,120]. Technical and procedure improvements are required to optimize the efficacy of these CT scans while minimizing the expenses associated with them.

EVs cannot multiply in the same way that cells do. As a result, uncontrolled cell multiplication can be avoided in this manner. This component of EV does not increase the likelihood of tumor growth. EVs are nano-sized vesicles that can enter tissues and even cells, as well as traverse the blood-brain barrier more easily than cells [102,121,122]. EVs can elude the immune system and, like cells, they have a negative zeta potential for extended circulation [123]. MSC-EVs confront several obstacles, including isolation, storage, and purification processes for MSC-EVs, which are always being revised and improved. Additionally, technical standards are being developed.

Engineered neurogenesis have been explored by various researchers by doping biomaterials in the form of nanoparticles, microspheres, nanotubes, micro-columns, and gels with cellular transplantation to support, enhance, and facilitate neurogenesis [124,125]. Poudel et al. elaborated the carrier materials [liposomes, micelles, solid lipid nanoparticles, polymeric nanoparticles, dendrimers, nanoemulsions, and inorganic nanoparticles] for neuronal regeneration in AD disease [126]. Bridget et al. reported that the cellular therapy offer an alternative approach for neurological repair and regeneration but the efficiency in regeneration remain low in both in vivo and in vitro models as injection. In an in vitro model of AD, Tween 80-methoxy poly(ethylene glycol)-poly(lactic-co-glycolic acid) nanoparticles incorporated with rhynchophylline resulted in increased delivery across the blood-brain barrier model which resulted in reduced cell death [127]. Researchers claimed that cellular transplantation in agarose micro-columns with an inner lumen filled with an appropriate extracellular matrix material regenerates neuronal cells [128]. Further research have to be explored on the ideal scaffold or carrier material for engineered neurogenesis for AD patients.

## 6 Conclusions

CT and or EVs have shown promising results in preventing the cognitive decline associated with AD in animal models which gives us more insight into the pathophysiology of the memory loss induced by the disease. Having known that the manifestations of the disease are not only due to the protein aggregates but also due to the neurogenic signal modulation, opens up the benefits of CT and EVs to patients with AD. MSCs and MSC-EVs seem to be the most practically plausible source without much ethical restrain in their usage thereby making it highly investigated for AD in the ongoing trials to translate their potential to clinical use to restore the lost memory due to AD.

## Funding

This research was supported by Basic Science Research Program through the 10.13039/501100003725National Research Foundation of Korea (NRF) funded by the 10.13039/100009950Ministry of Education (NRF-2022R1I1A1A01068652, NRF-2022R1I1A3068477 and NRF-2021R1I1A1A01040732).

## Author contribution statement

All authors listed have significantly contributed to the development and the writing of this article.

## Data availability statement

No data was used for the research described in the article.

## Declaration of competing interest

The authors declare that they have no known competing financial interests or personal relationships that could have appeared to influence the work reported in this paper
